# Behavioral and biochemical effects of alcohol withdrawal in female C3H/HeNRj and C57BL/6JRj mice

**DOI:** 10.3389/fnbeh.2023.1143720

**Published:** 2023-02-23

**Authors:** Simone Tonetto, Pia Weikop, Tomasz Brudek, Morgane Thomsen

**Affiliations:** ^1^Laboratory of Neuropsychiatry, Psychiatric Center Copenhagen, University Hospital of Copenhagen, Copenhagen, Denmark; ^2^Copenhagen Center for Translational Research, Bispebjerg-Frederiksberg Hospital, University Hospital of Copenhagen, Copenhagen, Denmark; ^3^Department of Neuroscience, Faculty of Health and Medical Sciences, University of Copenhagen, Copenhagen, Denmark; ^4^Center for Translational Neuromedicine, University of Copenhagen, Copenhagen, Denmark; ^5^Research Laboratory for Stereology and Neuroscience, Bispebjerg-Frederiksberg Hospital, University Hospital of Copenhagen, Copenhagen, Denmark

**Keywords:** alcohol withdrawal, mouse strains, behavioral tests, HPLC, neuroinflammation

## Abstract

**Background:**

Alcohol use disorder (AUD) is a major problem of our society and is often characterized and worsened by relapse. Prolonged alcohol exposure leads to numerous biochemical alterations that, upon cessation of alcohol intake, cause an array of immediate and lasting withdrawal symptoms. Acute withdrawal and neuroinflammation can be harmful in themselves, and lasting withdrawal symptoms contribute to relapse. Here, we conducted an initial feasibility study assessing several behavioral and neurochemical factors in female C3H/HeNRj (C3H) and C57BL/6JRj (B6) mice to determine which strain showed the clearest alcohol withdrawal symptoms during long-term abstinence and neurochemical alterations following re-exposure.

**Methods:**

Female C3H and B6 mice (*n* = 12 per group/strain) were intermittently exposed to alcohol-containing or control liquid diets for 3 weeks. Acute and prolonged withdrawal symptoms were assessed over a period of 3 weeks using a battery of behavioral test, comprised of alcohol self-administration, anhedonia, hyperalgesia, anxiety-like and depressive-like disturbances. Brain inflammation was measured by multiplex cytokine assay. Monoamine levels in the hippocampus and striatum, as well as exploratory analyses of cations levels in the cerebellum, were assessed by High-Performance Liquid Chromatography (HPLC).

**Results:**

Both C3H and B6 alcohol-exposed mice displayed decreased saccharin intake or preference and higher stress levels assessed by ultrasonic vocalizations (USVs) recordings. B6 but not C3H alcohol-exposed mice also exhibited a slower decline of alcohol oral self-administration (OSA), hyperalgesia, elevated brain TNF-α and elevated serotonin turnover.

**Conclusion:**

Our findings highlight the suitability of the B6 strain to study the behavioral and neurochemical alterations caused by alcohol withdrawal and the potential efficacy of experimental treatments, not only in early detoxification, but also in prolonged abstinence. The feasibility of these assays is important because long-lasting withdrawal symptoms are often the main cause of relapse in alcohol-dependent patients.

## 1. Introduction

Alcohol use disorder (AUD) is one of the most common psychiatric conditions, and more than 3 million deaths every year are linked to alcohol misuse worldwide (Hammer et al., 2018). Understanding the molecular mechanisms and correlations between neuroadaptive changes and withdrawal symptoms in alcohol dependence and relapse could lead to more effective interventions to tackle the global burden of harmful alcohol use.

Alcohol withdrawal syndrome is associated with a broad spectrum of neurobehavioral and neurochemical alterations, including irritability, dysphoria, sleep disturbances, anxiety, depression, delirium tremens and seizures, as well as neurochemical and neuroinflammatory changes. Although some of these symptoms present acutely and typically resolve within the first few days, negative emotional states can persist up to several weeks during prolonged abstinence, contributing to alcohol seeking and relapse (Engel et al., 2016; Hartmann et al., 2019). Previous rodent studies in alcohol addiction-related behaviors have looked at both the acute withdrawal phase, mainly looking at the somatic symptoms, and the long-lasting withdrawal symptoms (Heilig et al., 2010). It has been shown that both mice and rats display anxiety- and depressive-like behaviors up to several weeks after alcohol exposure, achieved either by vapor or liquid diet (Rylkova et al., 2009; Walker et al., 2010). These long-term withdrawal symptoms are believed to reflect neurocircuitry adaptations in multiple synaptic signaling systems, which can subsequently induce craving and relapse to overcome them (Koob and Le Moal, 2008).

Previous studies have described strain comparisons in alcohol-dependent rodents, including behavioral test series for alcohol withdrawal symptoms (Crabbe et al., 2005; Metten and Crabbe, 2005; Philip et al., 2010; Metten et al., 2018). Some of these reports described a clear withdrawal state in C3H mice, but focusing on the acute somatic withdrawal symptoms assessed by handling-induced convulsion (HIC) (Crabbe et al., 2005; Metten and Crabbe, 2005). Notably, these reports, including the direct comparison of C3H/HeNRj (C3H) and C57BL/6JRj (B6) mice in running wheel activity, used only male mice (Logan et al., 2012). Metten et al. (2018) recently established a series of behavioral tests in HS/Npt female and male mice, but mainly looking at the early withdrawal phase, in this case the first 98 h. They reported higher HICs in male mice compared to female mice and anhedonia only in males. Several withdrawal symptoms were manifested up to 3 weeks during alcohol abstinence in male B6 mice after alcohol binge drinking (Lee et al., 2015).

A clear neuroinflammatory pattern has been described in the serum of alcohol-dependent male patients (Yen et al., 2017). A similar pattern has also been shown in both serum and several brain regions of both female and male B6 mice, but no study has reported on C3H mice (Alfonso-Loeches et al., 2010; Pascual et al., 2015; Henriques et al., 2018). Differences in the cations content have also been reported in the serum of patients with AUD, although it remains to be shown whether serum levels correlated with or indeed are informative about brain levels. In particular, these individuals often showed hypomagnesemia and hypokalemia, as well as excessive serotonin (i.e., 5-hydroxytryptamine 5-HT), signaling, which has been implicated in the etiology of AUD and might play a role in increased alcohol craving in abstinent patients (Marcinkiewcz et al., 2016; Baj et al., 2020). Few studies reported similar outcomes in mice, including hypomagnesemia, hypophosphatemia and hypokalemia in the cerebellum and whole brain homogenates (Belknap et al., 1978; Te Riele and Verrips, 2014). Dopamine (DA), norepinephrine (NE) and 5-HT can all be altered in AUD and during withdrawal, with good concordance between human and rodent studies (Marcinkiewcz et al., 2016; Haass-Koffler et al., 2018; Grinevich et al., 2021). Conversely to a hyperdopaminergic state following acute alcohol exposure, chronic alcohol exposure and withdrawal result in a hypodopaminergic state that is thought to play a significant part in the neurotransmission mechanisms regulating alcohol dependence and relapse (Koob, 2003; Melis et al., 2005). Also in rats, DA levels in the nucleus accumbens (NAc) were reduced during withdrawal (Barak et al., 2011). Elevated NE levels were found both in the cerebrospinal fluid and plasma of AUD patients during early withdrawal (Hawley et al., 1981; Patkar et al., 2003). However, a report from Getachew et al. (2010), found decreased levels of DA, NE and 5-HT in the frontal cortex and hippocampus of rats during early withdrawal, associating the lower NE cortical levels to depressive like-symptoms observed in withdrawing rats. Overall, both animal and human studies have shown that dysregulation of the dopaminergic, noradrenergic and serotonergic signaling are key components of the pathophysiology of AUD and the mechanisms behind these neuroadaptive changes and their role in AUD need further investigation.

It is not evident which mouse strain is the most suitable for studies of alcohol withdrawal symptoms, thus additional studies are needed to investigate long-term withdrawal symptoms as well as the neurochemical adaptations involved. Moreover, the paucity of studies in female mice needs to be addressed, since both alcohol-dependent patients and mice display gender differences and women may experience more prolonged and severe long-term withdrawal symptoms than men (Finn et al., 2018; Navas et al., 2019; Wang et al., 2019). In the present study we examined multiple behavioral and biochemical endpoints in C3H and B6 female mice during a 3-week alcohol withdrawal window followed by 5 days re-exposure to determine which strain showed the most evident long-term withdrawal alterations. The C3H mouse strain was selected because it shows clear acute alcohol withdrawal symptoms (Metten and Crabbe, 2005; Logan et al., 2012). The B6 strain is a versatile and commonly used mouse strain that forms the basis for many research models (including many genetically altered lines available) and B6 male mice exposed to binge alcohol drinking displayed long-term alcohol withdrawal symptoms (Lee et al., 2015). We designed a battery of behavioral test, comprising alcohol self-administration, anhedonia, hyperalgesia, anxiety-like and depressive-like disturbances as well as specific assays for neuroinflammation and stress indicators to assess acute and more specifically long-term alcohol withdrawal symptoms during abstinence and following re-exposure.

## 2. Material and methods

### 2.1. Subjects

Twenty-four female C57BL/6JRj and 24 C3H/HeNRj mice (Janvier Labs, Le Genest Saint Isle, France) were acquired at 5–6 weeks of age and acclimated at least 1 week to the facilities before experimental procedures. Mice were group-housed 6 per cage (not necessarily litter mates) with hiding devices, nesting material, and wooden chewing blocks as enrichment, under a reversed 12 h light-dark cycle (light on 19:00–07:00). Tap water was available *ad libitum* throughout the study, and standard rodent chow (Altromin 1310, Brogaarden, Denmark) was available *ad libitum* until switching to liquid diet. Liquid diets were provided during experiments as described below. Procedures were approved by the Animal Experiments Inspectorate under the Danish Ministry of Food, Agriculture, and Fisheries in accordance with the EU directive 2010/63/EU.

### 2.2. Experiments and diets

**FIGURE 1:**
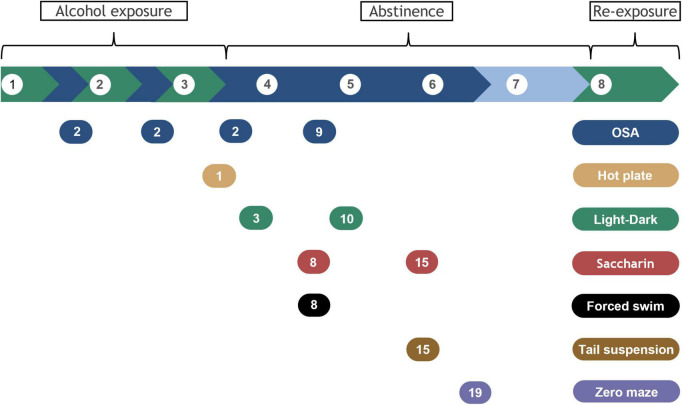
Timeline for alcohol exposure, abstinence, and behavioral assays. Alcohol exposure is highlighted in green and withdrawal periods in blue. Break phase is indicated in lighter blue. Brackets indicate alcohol exposure or abstinence phases and alcohol re-exposure before samples collection. Behavioral tests are indicated by the colored bubbles, with the testing day specified inside, counted as days of forced abstinence within each specific withdrawal period. White bubbles represent week number.

Alcohol dependence and withdrawal were induced using a liquid diet in 3 cycles of 5 days on 6.7% v/v alcohol, 2 days forced abstinence, as previously described (Figure 1; Bornebusch et al., 2021). After acclimation, mice were switched to liquid diets over 2 days (liquid diet with 3% alcohol + 3 g chow/mouse; then liquid diet with 4.5% alcohol + 1.5 g chow/mouse). Liquid diets with and without alcohol were isocaloric and were based on commercial rodent diets: the control diet consisted of 150.2 calories from fat, 659.9 from carbohydrates, and 190.2 from protein (per liter diet); the alcohol diet consisted of 150.5 calories from fat, 303.9 from carbohydrates, 189.9 from protein, and 356.3 from alcohol (per liter diet). After the third alcohol exposure cycle, alcohol was withheld for 3 weeks, and withdrawal symptoms were assessed using several assays described below. Finally, mice were re-exposed to alcohol for 5 days, then euthanized and blood and brains were collected as described below. For feasibility and internal replication, mice were tested in two consecutive cohorts balanced for strain and treatments (i.e., randomized block design). Mice were randomly assigned to one of two groups in each strain, control, and alcohol-exposed (*n* = 12 per group/strain). The experimenter was blinded to experimental group for all behavioral tests.

### 2.3. Operant oral alcohol self-administration (OSA)

Mice were trained to self-administer a 20% alcohol solution in operant-conditioning chambers (ENV-307A, Med Associates Inc., St Albans, VT, USA) prior to starting alcohol exposure. Briefly, the chambers contained two nose-poke holes, located on one wall of the chamber 3 cm above a grid floor, fitted with a photocell and a yellow cue light, and a steel dish into which reinforcers were delivered from a syringe pump (Thomsen and Caine, 2005; Bornebusch et al., 2019). All chambers were individually enclosed in sound-attenuating boxes equipped with a light and a ventilation fan. A nose-poke in the active hole triggered a syringe pump to deliver 30 μL of the reinforcer and the cue light to turn on. Nose-pokes in the inactive hole were recorded but had no scheduled consequences. Training sessions consisted of different solutions, starting with Nutridrink Compact Protein Vanilla (Nutricia, Amsterdam, Netherlands), then Calogen unflavored fat emulsion (Nutricia, Amsterdam, Netherlands), Calogen with 10% alcohol, Calogen with 20% alcohol and eventually water with 20% alcohol.

Training sessions used a fixed ratio (FR) 1 schedule of reinforcement, with a post-reinforcer timeout of 20 s, running for 1 h daily until criteria were met, i.e., a minimum of 20 reinforcers were earned within one session. Experimental sessions consisted of a progressive ratio (PR) schedule of reinforcement running for a maximum of 6 h per day. PR sessions terminated when no reinforcers were administered for 1 h (i.e., 1 h limited hold), at which time the number of reinforcers earned was recorded as the breaking point and the session ended. PR sessions consisted of a PR1 training period (response requirement 1, 2, 3 etc.), then, an exponential progression starting at 3 responses and increasing by a factor of 0.115 log units (response requirement 3, 4, 6, 7, 10, 13, 16, 21, 28, 36 etc.) which was used as the experimental schedule of reinforcement. The reinforcer-delivery cup was examined at the end of the sessions for any solution left unconsumed; in all the PR experimental sessions, all solution was consumed.

### 2.4. Hot-plate test

The hot-plate test, a supraspinal thermal pain assay, was used to measure pain sensitivity. Each mouse was placed on a horizontal surface (Hot Plate Analgesia Meter, Harvard Apparatus Limited, Holliston, MA, USA) preheated to 52°C and was confined to the plate with a tall Plexiglas cylinder. The testing room was set to low illumination (∼25 lux; Fisherbrand Traceable dual-range light meter, Traceable, Webster, TX, USA). The latency to first licking of hind paws or jumping was recorded. In the absence of a paw licking or a jumping response, a 60 s cut-off was used to prevent tissue damage.

### 2.5. Light-dark transition test

Light-dark transition test was conducted in open field activity chambers fitted with beam-break movement detection systems (OFA-510, Med Associates, St. Albans, VT, USA), as previously described (Bornebusch et al., 2021). A black plastic insert was used to create two compartments each measuring 27 × 13.5 cm, 30 cm tall, one dark and one with light. The light side had low illumination (∼40 lux) not anxiogenic alone (Hascoët et al., 2001), allowing detection of anxiogenic-like effects of alcohol withdrawal (Vranjkovic et al., 2018; McGinnis et al., 2020). The partition had a 4 × 4-cm opening allowing free movement between compartments. The voluntary time spent in the lit area, i.e., the total time spent in the lit area minus the latency to enter a dark area, and the total number of crossings between light and dark area were analyzed.

### 2.6. Saccharin preference test

The saccharin preference test was used to assess anhedonia. We used saccharin rather than sucrose to avoid potential confounders related to caloric content or diet composition, to make the test more widely applicable for future studies. A decrease in sucrose or saccharin intake and preference over water is generally taken as a putative sign of anhedonia in rodents (Strekalova and Steinbusch, 2010). Mice were tested individually in clean cages and given a free choice between two cups, one with 0.1% saccharin solution and another with tap water. The test took place in the dark. The liquid residues were measured after 4 h. Saccharin preference was calculated as saccharin intake divided by total volume of liquids consumed.

### 2.7. Forced swim test

Depressive-like effects were assessed using the Porsolt forced swim test, indicated by decrease in the latency to the first episode of immobility and an increase in the duration of the immobility time (Can et al., 2011b). Each mouse was placed in a Plexiglas cylinder, 20 cm in diameter and 50 cm in height, filled with lukewarm water (∼27°C) to a depth of 15 cm, so the mouse could not balance on its tail at the bottom of the cylinder or on the walls. The testing room illumination was set to ∼25 lux. The mice were tested and recorded for 6 min and then allowed to dry and rest in a separate cage placed on a preheated pad at 37°C. The duration of immobility was manually scored during the last 4 min of the trial. The latency to the first immobility episode was measured from the start. Immobility was regarded as the time the mouse spent floating in the water without any detectable movement, except those necessary to keep its head above water.

### 2.8. Tail suspension test

Depressive-like behavior was also assessed using the tail suspension test (Can et al., 2011c). Depressive-like behavior is exhibited by a decrease in the latency to the first episode of immobility and an increase in immobility time. Mice were suspended by taping their tails to a metal bar. The mice were positioned to preclude them from climbing or reaching nearby surfaces and from watching each other. A hollow cylinder 4 cm long was placed around the tail of all the B6 mice to prevent them climbing up their tail, which has been previously validated (Can et al., 2011a). The illumination was set to ∼25 lux. The test lasted 6 min and was recorded for later scoring. The latency to the first immobility bout and the duration of immobility were manually scored for the total duration of the test.

### 2.9. Elevated zero maze

Anxiety-like behaviors were also assessed with the elevated zero maze (Andreasen et al., 2015), considered to be an improvement of the elevated plus maze because it removes the ambiguity of the central area (Shepherd et al., 1994). It consists of an elevated ring-shaped platform, 50 cm above its base, with an inner diameter of 47 cm and two open and two closed zones with a 2.8 cm wide walking surface. The closed areas were surrounded by 11 cm high Plexiglas walls, and the open ones by a 6 mm edge. Each mouse was placed in the same closed area and its movements were recorded for 5 min. The testing room was set to ∼25 lux. The parameters were manually scored in real time and included the latency to first entry into an open area, the time spent on the open areas, the number of entries into the open areas as well as the number of stretched-attend postures (SAPs). A SAP is considered a risk assessment behavior, where a mouse stretches into an elongated posture, with its hind legs in the closed area and its fore legs in the open area. A mouse was considered as transitioning to an open area when all four legs completely passed from a closed to an open area.

### 2.10. Ultrasonic vocalizations (USVs)

It has been suggested that USVs in rodents reflect the stressful nature of withdrawal and the anticipatory positive affect of rewarding stimuli, including alcohol (Knapp et al., 1998; Buck et al., 2014). Ultrasonic calls were recorded with an Avisoft UltraSoundGate 416 Hb recording interface (Model CM16-CMPA, Avisoft Bioacoustics, Glienicke/Nordbahn, Germany) during the hot-plate test, the forced swim test, and in the first 10 min of the OSA. USV calls were recorded at a sampling rate of 250,000 Hz in 16 bit format. The recordings were analyzed using the Avisoft SASLab Pro software (Version 5.02.07, Avisoft Bioacoustics, Glienicke/Nordbahn, Germany), applying a fast fourier transformation (FFT) (1024 FTT length, 100% frame size, Blackman window and 87.5% time window overlap). Spectrograms were produced at a frequency resolution of 244 Hz and a time resolution of 0.512 ms. Spectrogram analysis was performed using an automatic whistle-tracking algorithm with an element separation threshold of −45 dB, 1 ms minimal duration and a hold time of 10 ms. USVs that were wrongly or not detected by the automatic whistle-tracking algorithm were manually assessed by an experienced observer. Various other spectrum-based parameters including peak frequency, peak amplitude and frequency modulation and temporal parameters such as call duration, total calling time and the total number of calls were also determined automatically.

### 2.11. Neuroinflammation assay

Brain samples from one hemisphere, except hippocampus, striatum and cerebellum, were dissected under microscope between 2 and 4 h after the last cycle of alcohol exposure, weighed and snap frozen at −80°C. Brain sample weighted around 100–130 mg and were transferred to MagNa Lyser tubes (F. Hoffmann-La Roche Ltd., Basel, Switzerland) containing 500 μL of tissue extraction reagent II (FNN0081, Thermo Fisher Scientific, Waltham, MA, USA) and 1x protease inhibitor cocktail (P8340, Merck KGaA, Darmstadt, Germany) and homogenized. The homogenization protocol consisted of two rounds of 25 s at 6000 rpm, with 90 s pause in ice. Samples were then spun at 16000 g at 4°C for 1 min to remove foam, transferred to Eppendorf tubes, and then centrifuged at 16000 *g* at 4°C for 20 min. Supernatant was aliquoted and frozen at −80°C. The total protein concentration was measured by Bradford assay (Merck KGaA, Darmstadt, Germany). The cytokine assay was performed using the ‘Mouse TH1/TH2 9-Plex Tissue Culture Kit’ [Meso Scale Diagnostics (MSD), Rockville, MA, USA], following manufacturer instructions. Brain samples were loaded 1:10 in phosphate buffer saline. The assay allowed detection of mouse interferon (IFN)-γ, interleukin (IL)-1β, IL-2, IL-4, IL-5, keratinocyte chemoattractant (KC)/human growth-regulated oncogene (GRO) also known as chemokine ligand 1 (CXCL1), IL-10, IL-12 (total), and tumor necrosis factor (TNF)-α with a dynamic range from 0.23 to 10000 pg/mL. Plates were read using MSD Sector Imager 6000 and data analyzed using MSD Workbench software. Final concentrations were adjusted to the brain sample weights and the protein concentrations.

### 2.12. High-Performance Liquid Chromatography (HPLC)

Hippocampus, striatum and cerebellum were dissected from one hemisphere under microscope between 2 and 4 h after the last cycle of alcohol exposure, weighed and snap frozen at −80°C. Hippocampus and striatum samples were each homogenized in 250 μL 0.1 M perchloric acid using an immersion hand disperser (Polytron PT 1200 E, Kinematica Inc., Keyland Court Bohemia, NY, USA). Cerebellum samples were homogenized with the same method in 500 μL milliQ water. Samples were then centrifuged at 14000 rpm at 4°C for 20 min and then supernatant collected using a 0.22 μm filter (Advantec, Sierra Court, CA, USA, 13CP020AS).

The concentration of the cations sodium (Na^+^), magnesium (Mg^++^), potassium (K^+^), and calcium (Ca^++^) in the cerebellum were determined by ion chromatography (IC, Dionex Aquion 1100, Thermo Fisher Scientific, Waltham, MA, USA). The cation chromatography consisted of a CS12A 4 mm analytical and a Dionex IonPac CG12A 4 mm guard column set. 20 mmol/L methanesulfonic acid was the eluent and was sonicated for 20 min followed by degassing with nitrogen for an additional 10 min prior to IC. 10 μl of sample were injected, was eluted for 20 min with isocratic 20 mmol/L methanesulfonic acid. Chromeleon Chromatography Data System software (Thermo Fisher Scientific, Waltham, MA, USA) was used to analyze and calculate the peaks.

The concentrations of norepinephrine (NE), 3,4-dihydroxy- phenylacetic acid (DOPAC), dopamine (DA), 5-hydroxy- indoleacetic acid (5-HIAA), homovanillic Acid (HVA) and serotonin (5-HT) in the striatum and hippocampus were determined by HPLC with electrochemical detection. The monoamines were separated by reverse-phase liquid chromatography with a Prodigy C18 column (DA 2 × 100 mm, particle size 3 μm, Phenomenex, YMC Europe, Schermbeck, Germany). The mobile phase (55 mM sodium acetate, 1 mM octanesulfonic acid, 0.1 mM Na2EDTA and 8% acetonitrile, adjusted to pH 3.7 with 0.1 M acetic acid) was de-gassed with an online de-gasser. 10 μl of sample were injected with a flow rate of 0.15 mL/min. Electrochemical detection was accomplished using an amperometric detector Antec Decade (Antec Scientific, Alphen aan den Rijn, Netherlands) with a glassy carbon electrode set at 0.8 V and an Ag/AgCl reference electrode. The output was recorded, and peak areas were calculated by LC solution software (Shimadzu, Kyoto, Japan).

### 2.13. Blood alcohol measurement

Trunk blood was collected 2–4 h after last exposure in EDTA-coated tubes on ice, centrifuged at 3000 rpm for 10 min and plasma was stored at −20°. Blood alcohol concentrations (BACs) were determined using a GL6 analyzer, Analox Instruments (Stokesley, UK).

### 2.14. Statistical analyses

A randomized block design was employed with two separate cohorts undergoing the exact same procedures. *A priori* power analyses were conducted using G*Power 3.1 to determine suitable sample sizes to detect effects of alcohol exposure/withdrawal in each strain, while strain differences *per se* were not an objective of the study. The error probability was set to 0.05 and power to 0.85. For most of the tests including the neuroinflammatory assay, power analyses based on published finding using similar methods (Alfonso-Loeches et al., 2010; Pascual et al., 2015; Metten et al., 2018) indicated *n* = 6/group. However, for the forced swim and hot plate tests, analyses indicated *n* = 7 and *n* = 30, respectively (Karadayian et al., 2013; Lee et al., 2015). We selected *n* = 12 to allow detection of smaller effects (e.g., later in withdrawal) with the caveat that the hot-plate test may be underpowered.

Statistical analyses were performed using InVivoStat (Version 4.6.0) and R (version 3.6.3). All tests were performed with cohort as a blocking factor, when applicable. Bodyweight was analyzed by mixed model repeated measures (MMRM), with time as repeated factor and treatment as between-subject factor, followed by planned multiple comparison adjustments using false discovery rate *via* Benjamini–Hochberg’s procedures (alcohol vs. control for each withdrawal day). Alcohol intake from diets was also analyzed by MMRM, with time as repeated factor and strain as between-subject factor to confirm differences previously reported, followed by multiple comparison adjustments using false discovery rate *via* Benjamini–Hochberg’s procedure. For the OSA data, a linear curve over time was fitted to each animal, followed by one-way analysis of co-variance (ANCOVA) on the intercept and slope parameters with baseline as the covariate and cohort as a blocking factor. Light-dark, tail suspension, and elevated zero maze data were analyzed by one-way analysis of variance (ANOVA) with treatment as the between-subject factor. Saccharin preference and forced swim tests were evaluated by unpaired *t*-test since only one cohort was analyzed and thus there was no need for a blocking factor. Latencies to events were analyzed by cox proportional hazards regression model with cohort as a blocking factor. HPLC and neuroinflammatory data were analyzed by MMRM with marker (cytokines, monoamines, or cations) as the repeated factor, treatment as the between-subject factor, and cohort as a blocking factor, followed by planned multiple comparison adjustments using false discovery rate *via* Benjamini–Hochberg’s procedures. Neuroinflammatory data was square root transformed because it did not satisfy the parametric analysis assumptions and assessed by a compound symmetric covariance structure. HPLC data was log(10) transformed because it did not satisfy the parametric analysis assumptions and assessed with an unstructured covariance structure. HPLC datapoints were considered as outliers when standard deviation differed more than four times from the group mean. HPLC ratios were analyzed individually by a one-way ANOVA, with cohort as a blocking factor. Graphs were plotted using Prism (Version 9, GraphPad, San Diego, CA, USA). Alcohol intake and OSA as well as bodyweight data are shown as group means ± SEMs. All other data are presented as individual subjects, either with violin plots representing group means, distribution, and density or as survival curves. Differences are reported as significant for *p*-values < 0.05.

## 3. Results

### 3.1. Diet intake and bodyweight

**FIGURE 2:**
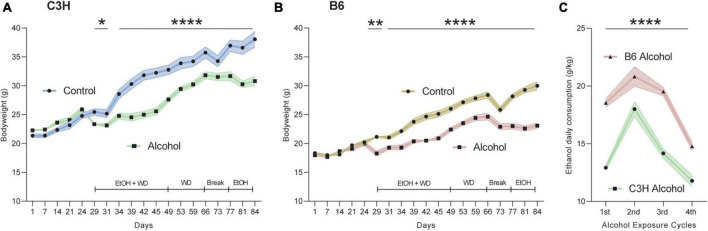
Bodyweight and alcohol intake over time. Bodyweight in grams during the whole experiment for C3H **(A)** and B6 **(B)** mice. Alcohol intake as g/kg/day over the four exposure periods for C3H and B6 mice **(C)**. Data are shown as group means, with the shaded area representing ± SEMs. *n* = 12. ****p < 0.0001, **p < 0.01, and *p < 0.05 represent mixed model repeated measures (MMRM) followed by Benjamini–Hochberg’s *post hoc*, alcohol vs. control **(A,B)** and C3H vs. B6 **(C)**.

Both mouse strains were similarly affected by the liquid diets containing alcohol, showing moderately decreased bodyweight gains throughout the whole experiment compared to the control diet. Indeed, MMRM analyses revealed a significant effect of treatment (*F*_1,21_ = 15.4, *p* = 0.0008), time (*F*_18,396_ = 311.3, *p* < 0.0001) and treatment-time interaction (*F*_18,396_ = 35.1, *p* < 0.0001) for the C3H mice (Figure 2A). Similarly, the B6 exhibited a significant effect of treatment (*F*_1,21_ = 39.5, *p* < 0.0001), time (*F*_18,396_ = 261.6, *p* < 0.0001) and treatment-time interaction (*F*_18,396_ = 30.5, *p* < 0.0001) (Figure 2B). B6 mice consumed more alcohol compared to their C3H counterpart, supported by a significant effect of strain (*F*_1,21_ = 118.0, *p* < 0.0001) and strain-time interaction (*F*_3,66_ = 5.60, *p* = 0.002) (Figure 2C). *Post hoc* comparisons revealed a significantly higher daily alcohol consumption in B6 compared to C3H at each of the 4 weeks of exposure (*p* < 0.0001, except *p* = 0.0001 for week 2) (Figure 2C). Due to a technical issue, BACs were measured only in the second cohort and did not differ between the two strains (51.07 ± 3.09 mg/dL for C3H and 61.02 ± 4.03 mg/dL for B6).

### 3.2. Oral alcohol self-administration

Both C3H and B6 mice showed some significant differences in oral alcohol self-administration between alcohol-exposed and control mice, as measured by the number of reinforcers taken (i.e., breaking point) and by number of active nose-pokes over the entire observation period.

**FIGURE 3:**
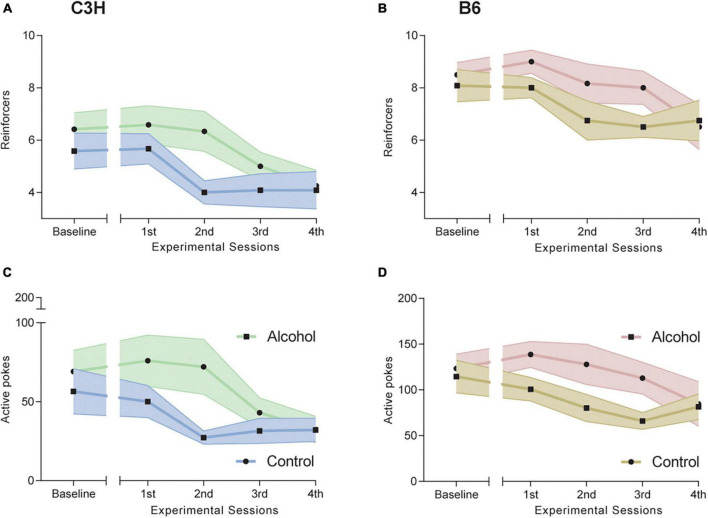
Effects of alcohol exposure and subsequent withdrawal in alcohol oral self-administration (OSA). Number of reinforcers taken **(A,B)** and of active nose pokes **(C,D)** during the five progressive ratio (PR) experimental sessions for C3H **(A,C)** and B6 **(B,D)** mice. Data are shown as group means, with the shaded area representing ± SEMs. *n* = 12 per group/strain.

C3H mice had a trend toward steeper slope (−15.99 ± 1.33 vs. −4.96 ± 0.79) and higher intercept (111.87 ± 6.91 vs. 52.63 ± 3.67) in alcohol-exposed mice for active pokes (*F*_1,20_ = 3.5, *p* = 0.078 and *F*_1,20_ = 4.3, *p* = 0.051), but not for reinforcers taken (Figures 3A, C). ANCOVAs for B6 mice showed significantly higher intercept (178.04 ± 7.15 vs. 107.15 ± 3.03) and steeper slope (−17.71 ± 1.94 vs. −7.15 ± 0.9) for active pokes in alcohol-exposed B6 mice (*F*_1,19_ = 8.1, *p* = 0.01 and *F*_1,20_ = 7.1, *p* = 0.015), and higher intercept (10.6 ± 0.18 vs. 8.4 ± 0.11) for reinforcers taken (*F*_1,20_ = 8.8, *p* = 0.0075) (Figures 3B, D). The slope and intercept define the linear relationship between either the number of reinforcer or active pokes and time, estimating a rate of change.

### 3.3. Hot plate

**FIGURE 4:**
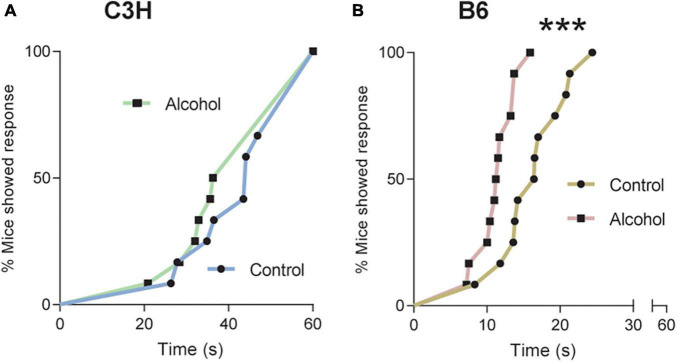
Pronociceptive effects of alcohol exposure and subsequent withdrawal. Latencies in seconds to nociceptive response in C3H **(A)** and B6 **(B)** mice tested on a 52°C hot-plate at withdrawal day 1, with a cutoff at 60 s. Data are shown as the cumulative% of mice showing a response as a function of time. Multiple mice with the same latency appear as individual points. Note the truncated abscissa in panel **(B)**. *n* = 12. ***p < 0.001 represents cox proportional hazard regression, alcohol vs. control.

Cox proportional hazard regression revealed no significant difference between the two groups of C3H mice on day 1 of withdrawal (Figure 4A). However, B6 mice exposed to alcohol displayed significantly reduced latency to first licking of hind paws or jumping compared to the controls (*p* = 0.0007; Figure 4B).

### 3.4. Light-dark

**FIGURE 5:**
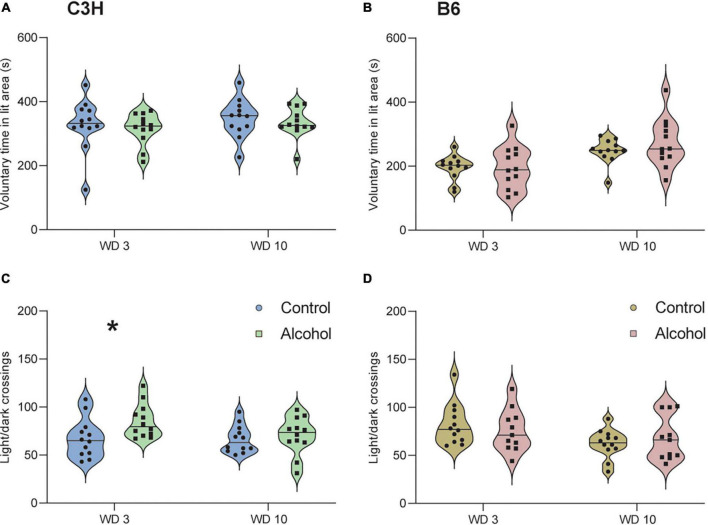
No anxiogenic-like effects of alcohol exposure and subsequent withdrawal detected in the light/dark test. The % of time spent voluntarily in the lit area **(A,B)** and the number of light/dark crossing **(C,D)** were assessed in C3H and B6 mice at the 3rd and 10th day of alcohol withdrawal. Data are shown as individual subjects, horizontal line in violin plots represents group means. *n* = 12 per group/strain. *p < 0.05 represents One-way ANOVA followed by Benjamini–Hochberg’s *post hoc*, alcohol vs. control.

The two timepoints were analyzed separately, since it is known that mice can habituate to anxiogenic effects in the test (Hascoët et al., 2001). One-way ANOVA (*F*_1,21_ = 5.5, *p* = 0.03) revealed significantly more light/dark crossings in C3H mice exposed to alcohol relative to control diet on withdrawal day 3, but no difference on withdrawal day 10 (*F*_1,21_ = 0.3, *p* = 0.6) (Figure 5C). There were no significant differences for voluntary time spent in the light area in C3H mice, either on day 3 or 10 of withdrawal (*F*_1,21_ = 0.3, *p* = 0.6 and *F*_1,21_ = 0.3, *p* = 0.6) (Figure 5A). There were no significant differences either in voluntary time spent in the light area (*F*_1,21_ = 0, *p* = 0.98 and *F*_1,21_ = 1.5, *p* = 0.2) or in the number of light/dark crossing (*F*_1,21_ = 0.3, *p* = 0.6 and *F*_1,21_ = 0.7, *p* = 0.4) in B6 mice, either on day 3 or 10 of withdrawal (Figures 5B, D).

### 3.5. Saccharin preference test

**FIGURE 6:**
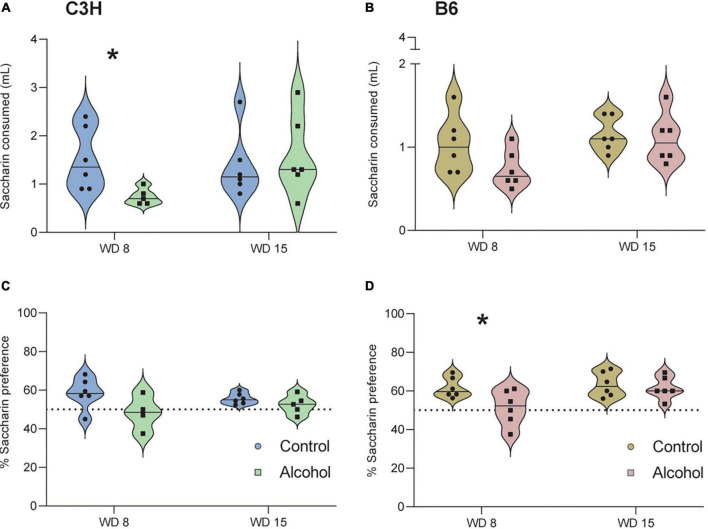
Anhedonia-like effects following alcohol exposure and subsequent withdrawal in the saccharin preference test. The saccharin intake **(A,B)** and the % of saccharin preference **(C,D)** were assessed in C3H and B6 mice at the 8th and 15th day of alcohol withdrawal. Data are shown as individual subjects with violin plots. Note the truncated ordinate in panel **(B)**. *n* = 4–6 per group/strain. *p < 0.05 represents unpaired *t*-test, alcohol vs. control.

Only data from the second cohort were analyzed, because the duration of the test was extended from 2 to 4 h. The two timepoints were analyzed separately, since it is known that mice can habituate (Strekalova and Steinbusch, 2010; Liu et al., 2018). C3H mice exposed to alcohol showed a significantly lower saccharin intake (*p* = 0.03; Figure 6A) and a trend toward decreased saccharin preference (*p* = 0.09; Figure 6C) on day 8 of withdrawal, relative to the controls. Similarly, B6 mice exposed to alcohol showed a trend for reduced saccharin intake on day 8 of withdrawal (*p* = 0.1; Figure 6B), and a significantly lower saccharin preference (*p* = 0.04; Figure 6D). Neither strain exhibited an effect of alcohol exposure on saccharin preference (C3H *p* = 0.2, B6 *p* = 0.6) and saccharin intake (C3H *p* = 0.7, B6 *p* = 0.7) on day 15 of withdrawal.

### 3.6. Forced swim test

Only data from the second cohort were analyzed, because the diameter of the cylinder was changed from 10 to 20 cm. No significant differences were found in the C3H or B6 mice regarding the % of time spent immobile (C3H *p* = 0.2, B6 *p* = 0.6) or the latency to first immobility (C3H *p* = 0.09, B6 *p* = 0.4) (Supplementary Figures 1A–D).

### 3.7. Tail suspension test

**FIGURE 7:**
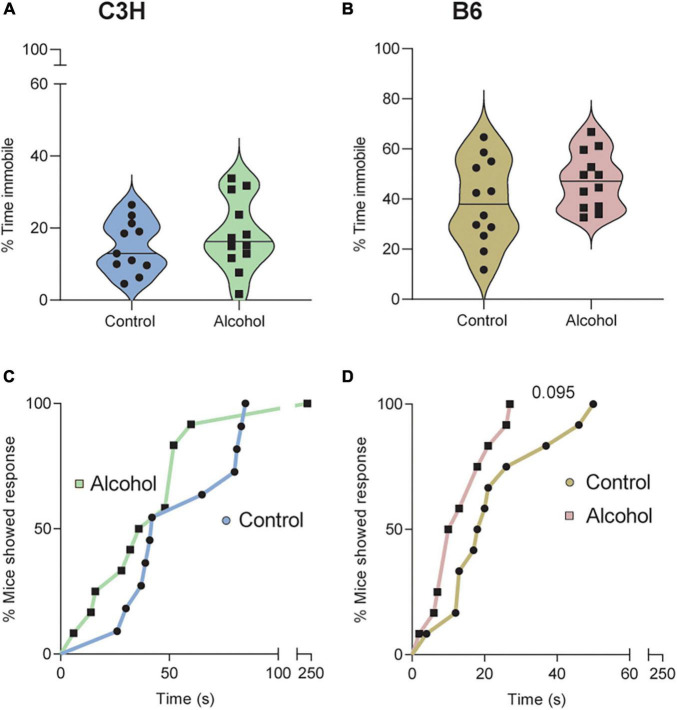
Trend for depressive-like effects of alcohol exposure and subsequent withdrawal in the tail suspension test. The % of time spent immobile **(A,B)** and the latencies in seconds to first immobility **(C,D)** were assessed in C3H and B6 mice. Data are shown as individual subjects with violin plots **(A,B)**. Latencies are shown as the % of mice showing a response as a function of time **(C,D)**. Note the truncated ordinate in panel **(A)** and truncated abscissa in panels **(C,D)**. *n* = 12 per group/strain.

No significant treatment effects were found in the C3H or B6 mice regarding the % of time spent immobile (*F*_1,20_ = 0.9, *p* = 0.4 and *F*_1,21_ = 2.3, *p* = 0.1) on day 8 of withdrawal (Figures 7A, B). Data suggested increased time spent immobile in the B6 mice exposed to alcohol (Figure 7B), but this did not reach statistical significance (*p* = 0.14). Cox proportional hazards regression model revealed a trend toward lower latency to first immobility in the B6 mice exposed to alcohol (*p* = 0.095; Figure 7D), but not in the C3H mice (*p* = 0.42; Figure 7C).

### 3.8. Elevated zero maze

Statistical analyses revealed no significant differences in either mouse strain between alcohol exposed and control groups on day 19 of withdrawal, for several parameters, including the time spent on the open areas (C3H *F*_1,14_ = 0.4, *p* = 0.5 and B6 *F*_1,21_ = 0.2, *p* = 0.6), the number of entries into the open areas (C3H *F*_1,21_ = 0.8, *p* = 0.4 and B6 *F*_1,21_ = 0.9, *p* = 0.4), the number of SAPs (C3H *F*_1,21_ = 2.1, *p* = 0.2 and B6 *F*_1,21_ = 2, *p* = 0.2) and the latency to first entry into an open area (C3H *p* = 1 and B6 *p* = 0.3) (Supplementary Figures 2A–H).

### 3.9. USVs

No USVs were recorded during the hot plate test or the first 10 min of the OSA. However, two C3H mice and two B6 mice exposed to alcohol were the only mice to emit USVs, both low and high frequency calls (<60 and >60 kHz, respectively) during the forced swim test, while no control mice produced any USVs. One C3H mouse emitted a single high frequency call and the other C3H mouse emitted a single high and a single low frequency call. One B6 mouse emitted eight high frequency calls and two low one, whereas the other B6 mouse emitted two low frequency calls (no further data detail shown).

### 3.10. Neuroinflammation

**FIGURE 8:**
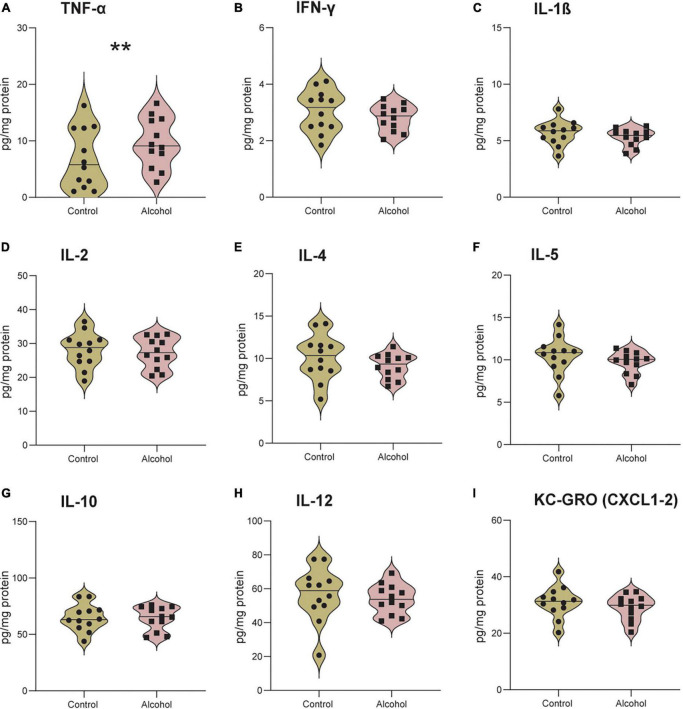
Increased neuroinflammation following alcohol exposure in B6 mice. The brain levels of TNF-α **(A)**, IFN-γ **(B)**, IL-1 β **(C)**, IL-2 **(D)**, IL-4 **(E)**, IL-5 **(F)**, IL-10 **(G)**, IL-12 **(H)**, and KC-GRO **(I)** in B6 mice. Data are shown as individual subjects with violin plots. *n* = 12 per group/strain. **p < 0.01 represents mixed model repeated measures (MMRM) followed by Benjamini–Hochberg’s *post hoc*, alcohol vs. control.

MMRM analyses of B6 data revealed a significant effect of treatment-marker interaction (*F*_8,176_ = 2.9, *p* = 0.004) but no main effect of treatment (*F*_1,21_ = 0.4, *p* = 0.9). *Post hoc* analysis of the interaction revealed significantly higher levels of TNF-α in B6 mice exposed to alcohol compared to controls (*p* = 0.005), but no difference for the other cytokines/chemokines (Figures 8A–I).

**FIGURE 9:**
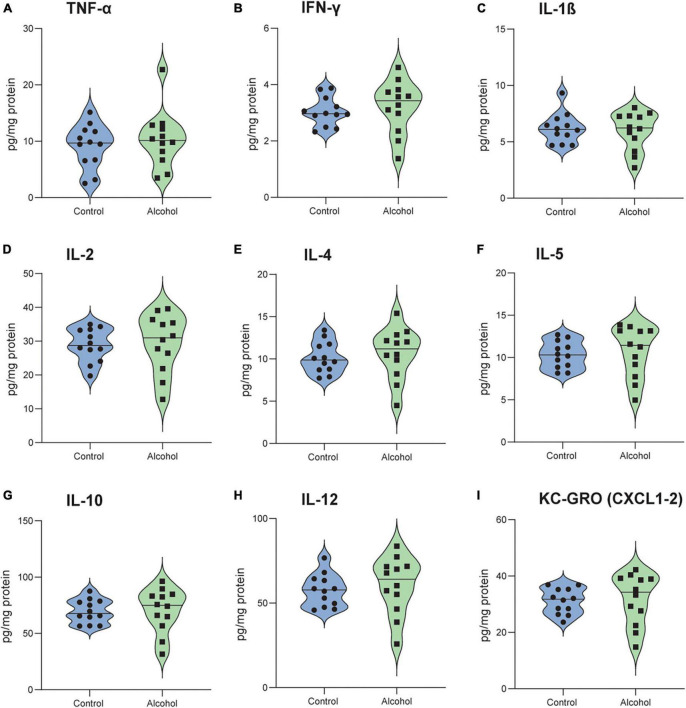
No detectable neuroinflammation following alcohol exposure in C3H mice. The brain levels of TNF-α **(A)**, IFN-γ **(B)**, IL-1 β **(C)**, IL-2 **(D)**, IL-4 **(E)**, IL-5 **(F)**, IL-10 **(G)**, IL-12 **(H)** and KC-GRO **(I)** in C3H mice. Data are shown as individual subjects with violin plots. *n* = 12 per group/strain.

No significant effects of either treatment or treatment-marker interaction (*F*_1,21_ = 0.2, *p* = 0.6 and *F*_8,176_ = 0.5, *p* = 0.9) were detected between C3H mice exposed to alcohol and controls (Figures 9A–I).

### 3.11. Brain biochemistry

**FIGURE 10:**
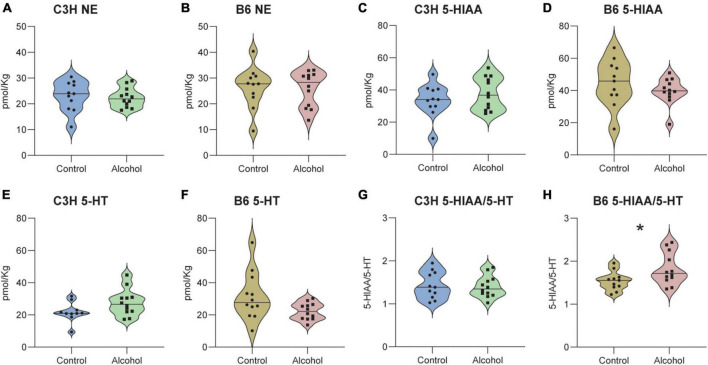
Neurotransmitter levels following alcohol exposure in the hippocampus of C3H and B6 mice. The levels of norepinephrine (NE) **(A,B)**, 5-HIAA **(C,D)**, 5-HT **(E,F)**, and 5-HIAA/5-HT **(G,H)** in the hippocampus of C3H and B6 mice. Data are shown as individual subjects with violin plots. *n* = 11–12 per group/strain. *p < 0.05 represents mixed model repeated measures (MMRM) followed by Benjamini–Hochberg’s *post hoc*, alcohol vs. control.

**FIGURE 11:**
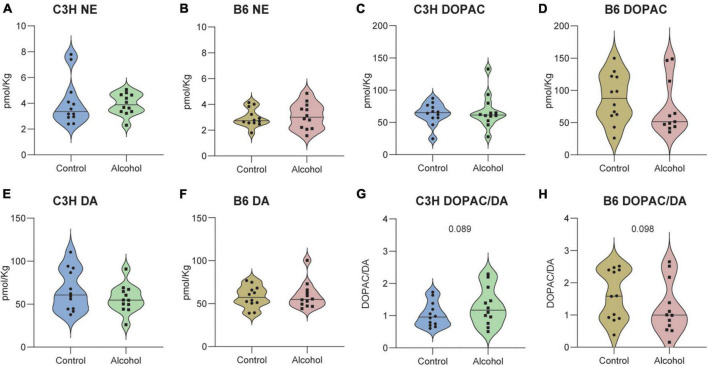
Neurotransmitter levels following alcohol exposure in the striatum of C3H and B6 mice. The levels of norepinephrine (NE) **(A,B)**, DOPAC **(C,D)**, DA **(E,F)**, and DOPAC/DA **(G,H)** in the striatum of C3H and B6 mice. Data are shown as individual subjects with violin plots. *n* = 11–12 per group/strain.

No significant effects of treatment (C3H *F*_1,20_ = 0.6, *p* = 0.5 and B6 *F*_1,21_ = 1.4, *p* = 0.3) or treatment-marker interaction (C3H *F*_2,42_ = 1, *p* = 0.4 and B6 *F*_2,44_ = 1.2, *p* = 0.3) were detected in the concentrations of NE, 5-HIAA, and 5-HT in the hippocampus of C3H and B6, and no significant treatment effect for 5-HIAA/5-HT ratio (*F*_1,20_ = 0.1, *p* = 0.8) in the C3H (Figures 10 A–G). B6 alcohol-exposed mice had higher serotonin turnover in the hippocampus, as suggested by the increased 5-HIAA/5-HT (*F*_1,20_ = 7.3, *p* = 0.01) (Figure 10H). MMRM revealed no significant effects of treatment (C3H *F*_1,21_ = 0.05, *p* = 0.8 and B6 *F*_1,21_ = 0.05, *p* = 0.8) or treatment-marker interaction (C3H *F*_5,103_ = 0.9, *p* = 0.5 and B6 *F*_5,84_ = 1.1, *p* = 0.4) for the concentrations of NE, DOPAC, and DA in the striatum of C3H and B6 (Figures 11A–F). Interestingly, C3H alcohol-exposed mice showed a trend toward higher dopamine turnover in the striatum, measured as the DOPAC/DA ratio (*F*_1,21_ = 3.2, *p* = 0.089), whereas B6 alcohol-exposed mice displayed a trend toward lower dopamine turnover (*F*_1,21_ = 3, *p* = 0.098) (Figures 11G, H).

**FIGURE 12:**
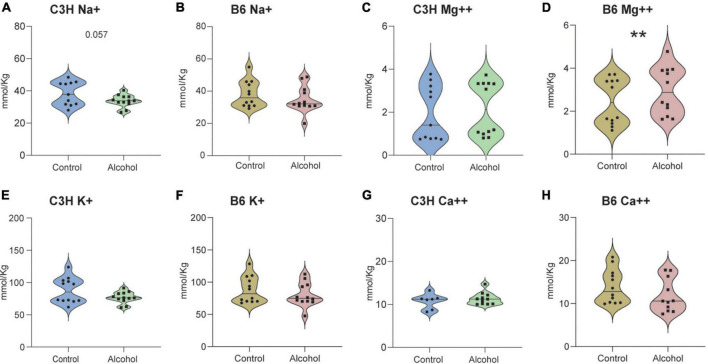
Cation alterations following alcohol exposure in the cerebellum of C3H and B6 mice. The concentration of cations Na^+^ **(A,B)**, Mg^++^ **(C,D)**, K^+^ **(E,F)**, Ca^++^ **(G,H)** in the cerebellum of C3H and B6 mice. Data are shown as individual subjects with violin plots. *n* = 8–12 per group/strain. **p < 0.01 represents mixed model repeated measures (MMRM) followed by Benjamini–Hochberg’s *post hoc* alcohol vs. control.

Significant alterations were found in the cation content in the cerebellum of C3H and B6 alcohol-exposed mice compared to controls (Figures 12 A–H). MMRM analyses revealed a trend for a treatment-marker interaction (*F*_3,61_ = 2.2, *p* = 0.09) but no main effect of treatment (*F*_1,21_ = 2.8, *p* = 0.1) in C3H mice. Multiple comparisons revealed a trend toward decreased levels of Na^+^ (*p* = 0.057) in the cerebellum of C3H alcohol-exposed mice (Figure 12A). MMRM analyses of B6 data revealed a significant treatment-marker interaction (*F*_3,65_ = 5, *p* = 0.004), but no main effect of treatment (*F*_1,21_ = 0.2, *p* = 0.6). Multiple comparisons revealed increased levels of Mg^++^ (*p* = 0.0016) in the cerebellum of B6 alcohol-exposed mice (Figure 12D).

## 4. Discussion

Neuropsychiatric disturbances are among the most subtle and complex symptoms in AUD. Often, these last for a long period during abstinence, eventually causing relapse. Here, we conducted a strain comparison to find the most suitable mouse strain to investigate candidate treatments, while also establishing the feasibility of a series of long-term behavioral and biochemical tests. Previous studies have described a more pronounced acute somatic withdrawal state in C3H/HeJ (The Jackson Laboratory; Bar Harbor, ME) mice relative to B6 mice, measured by HIC (Crabbe et al., 2005; Metten and Crabbe, 2005). In contrast, Metten et al. (2010) reported few HIC for C3H/HeJ compared to B6 mice. It is important to note that these studies reporting contrasting results, used the same C3H/HeJ substrain from The Jackson Laboratory, whereas Becker et al. (1997) used a different substrain, C3H/HeNCrl from Charles River Laboratories (Portage, MI, USA), manifesting substantial acute withdrawal assessed by HIC (Veatch et al., 2007). Interestingly, it has being discovered that the C3H/HeJ substrain developed a spontaneous mutation in the toll-like receptor 4 gene, which plays an important role in inflammation and could have an impact on withdrawal symptoms. In this initial feasibility study, female C3H/HeNRj and C57BL/6JRj mice from Janvier Labs were used. The B6 mice showed clear alcohol withdrawal symptoms, both in terms of behavioral and biochemical alterations: increased pain sensitivity, slower reduction in alcohol OSA, decreased saccharin preference, a tendency toward a depressive-like state, as well as increased TNF-α levels and serotonin turnover. The C3H mice showed less evident symptomatology, characterized by decreased cation levels in the cerebellum, decreased saccharin intake, a tendency toward slower reduction in alcohol OSA and toward a depressive-like state. The results are summarized in Table 1.

**TABLE 1:** Summary of behavioral and biochemical effects in C3H and B6 mice.

	C3H	B6
OSA	(+)	+
Hyperalgesia	N/E	+
Anxiety-like symptoms	–	N/E
Depressive-like symptoms	+	+
USVs	(+)	(+)
Neuroinflammation	N/E	+
Monoamine changes	(+)	(+)
Cation imbalances	(−)	+

+ denotes a significant increased effect and − denotes a significant reduction; (+) denotes a trend or mixed results and (−) denotes a trend toward lower levels; N/E denotes no effect.

Both C3H and B6 alcohol-exposed mice displayed a slower reduction in alcohol OSA during the withdrawal periods compared to control mice, with a clearer pattern in the B6 mice (i.e., reaching statistical significance). It has been previously described that mice escalated their alcohol self-administration following chronic alcohol exposure, including using exposure by vapor inhalation, liquid diets and intragastric infusion (Chu et al., 2007; Finn et al., 2007; Cunningham et al., 2013; Lopez and Becker, 2014; Sneddon et al., 2020). One of the reasons why we did not observe an escalation might be due to the lower BACs reached in this experiment. Indeed, Griffin et al. (2009) stated that the escalation effect is determined by repeated cycles of chronic intermittent alcohol exposure, producing sustained BACs of at least 175 mg/dL. Another explanation could be the self-administration protocol used in this study, which consisted of a steeply incrementing PR schedule with a maximum duration time of 6 h, yielding a low density of alcohol reinforcers per time and effort. It is possible that different schedules of reinforcement better reveal different aspects of alcohol withdrawal, such as effort willingness vs. rewarding effects. Every self-administration report mentioned used solely male mice and FR 2/3/4/10, whereas the present study used female mice. One could hypothesize that male mice might sensitize to alcohol self-administration following repeated cycles of alcohol exposure but females might not, like Veatch et al. (2007) described in the case of HIC.

B6 mice exposed to alcohol displayed a reduced latency to lick the hind paws or jump in the hot plate test, a clear sign of increased pain sensitivity, measured on day one of withdrawal. Although the power analysis indicated large groups (*n* = 30) may be needed to reliably capture alcohol withdrawal effects, we believe the present results, collected from two independent randomized cohorts, suggest a clear pattern of hyperalgesia in B6 alcohol-exposed mice. Previous studies described the role of alcohol dependence as a chronic pain disorder in humans (Egli et al., 2012; Edwards et al., 2020). However, we acknowledge that this type of assay may yield variable results, and indeed there is contrasting evidence in different mouse models, with male Swiss mice and B6 mice manifesting increased pain sensitivity, whereas HS/Npt mice were not affected (Karadayian et al., 2013; Alongkronrusmee et al., 2016; Metten et al., 2018). This further underlines the importance of strain differential susceptibility to alcohol withdrawal symptoms.

Neither B6 nor C3H mice showed increased anxiety-like behavior when measured with the light/dark test or the elevated zero maze. Surprisingly, C3H alcohol-exposed mice showed increased light-dark crossing on the third withdrawal day. Lee et al. (2015) measured anxiety-like behaviors on day 1 and day 21 of withdrawal in male C57BL/6J mice, observing greater hyperactivity in the light side and fewer light-side entries in alcohol-exposed mice compared to controls at both time points. However, the authors of that study do not mention the light intensity used in the light/dark test, which is a critical parameter and could explain the different results. Other studies reported reduced voluntary time in the lit side and reduced light/dark crossings in the first withdrawal day (Kliethermes, 2005; Bornebusch et al., 2021). The elevated zero maze also failed to reveal any significant differences in the anxiety-like behavior of both mouse strains. We hypothesize that we were not able to observe these anxiety-like behaviors because the first light/dark test was performed on day 3 of withdrawal and the zero maze on day 19 of withdrawal, possibly too late to detect any effects. Alternatively, withdrawal symptoms were not as strong due to the use of liquid diets rather than vapor. Indeed, some previous studies showed clear evidence that alcohol vapor-exposed male Swiss, male B6 and HS/Npt mice displayed anxiety-like features in the elevated zero maze and also in the elevated plus maze, both in the early and late withdrawal phases (Karadayian et al., 2013; Lee et al., 2015; Metten et al., 2018).

Alcohol-exposed B6 mice showed significantly decreased saccharin preference on day 8 of withdrawal only, while alcohol-exposed C3H mice exhibited a similar trend and a significantly lower saccharin intake on day 8. Metten and collaborators observed decreased preference for saccharin in alcohol-exposed HS/Npt mice on day 3 and 4 of withdrawal (Metten et al., 2018). However, another study reported no effect in the first withdrawal day in male Swiss mice, but these mice were treated with intraperitoneal alcohol injections for only 6 h (Karadayian et al., 2013). Lee et al. (2015) also did not observe any effect in an overnight saccharin preference test on days 1 and 21 of withdrawal, with C57BL/6J male mice exposed to alcohol *via* a drinking-in-the-dark procedure. Thus, it appears that alcohol-exposed mice show some signs of anhedonia during the early withdrawal, but these can be subtle and difficult to detect. The method and duration of alcohol exposure and the duration of the saccharin preference test play a critical role in detecting this symptom, especially if assessed in the long-term withdrawal. No significant differences were detected in the forced swim test in our study. In contrast, previous evidence indicated that mice spent more time immobile and had a shorter latency to first immobility, both in the early and late phases of withdrawal (Karadayian et al., 2013; Lee et al., 2015). In the present study, only B6 mice displayed a trend toward increased time spent immobile and decreased latency to first immobility in the tail suspension test, on day 15 of withdrawal. Karadayian et al. (2013) reported significantly increased time spent immobile and decreased latency to first immobility in the tail suspension test in male Swiss mice, on day 1 of withdrawal.

Two C3H and two B6 alcohol-exposed mice emitted USVs during the forced swim test, while no control mice produced any USVs. This could be a symptom of distress in alcohol-exposed mice, but USVs may be a less robust method for studying withdrawal-associated affective states in mice than in rats, in which most previous studies were conducted. Indeed, we found no reports on UVSs emission in adult mice during alcohol withdrawal. A previous study reported association of anticipatory 50 kHz USVs with escalated alcohol intake in dependent rats (Buck et al., 2014). Our findings suggest that increased USV emissions by alcohol-exposed mice during withdrawal could be an important symptom of a general negative emotional state, but further studies are required to extend the recording to other tests and refine methods.

Importantly, only B6 mice exposed to alcohol displayed increased levels of brain TNF-α. Previous evidence described a similar neuroinflammatory pattern in the striatum, cortex and serum of B6 mice, in both females and males (Alfonso-Loeches et al., 2010; Pascual et al., 2015; Henriques et al., 2018). Moreover, Yen et al. (2017) reported increased levels of both pro- and anti-inflammatory cytokines in the plasma of alcohol-dependent patients during the early withdrawal state, which decreased after 4 weeks of abstinence but still remain significantly different compared to controls. Here we report for the first time that C3H female mice do not show a detectable neuroinflammatory pattern under conditions that allowed its detection in B6 mice. Even though only TNF-α levels were increased in B6 alcohol-exposed mice and no differences were found in the other cytokines/chemokines, TNF-α is one of the hallmarks of neuroinflammation in alcohol-exposed rodents (Henriques et al., 2018). Moreover, most of the studies measured neuroinflammation in specific brain regions and/or serum, whereas we investigated overall brain levels.

Despite extensive evidence on the dysregulation of dopaminergic, noradrenergic, and serotonergic signaling in AUD, we detected no significant changes in the concentrations of NE, DA, 5-HT, HVA, and 5-HIAA in both the striatum and hippocampus of C3H and B6 mice (Fitzgerald, 2013; Marcinkiewcz et al., 2016; Haass-Koffler et al., 2018; Khom et al., 2020; Grinevich et al., 2021). Notably, B6 alcohol-exposed mice displayed higher serotonin turnover in the hippocampus, as suggested by the increased 5-HIAA/5-HT ratio, measured indirectly, suggesting an alteration of the serotoninergic signaling. Both mouse strains also exhibited a trend toward altered dopamine turnover, measured by the DOPAC/DA ratio; C3H alcohol-exposed mice showed higher dopamine turnover, whereas B6 alcohol-exposed mice showed a lower dopamine turnover, in agreement with a previous report (Boutros et al., 2018). In this study the chronic alcohol exposure was followed by a long-term abstinence of 3 weeks and eventually by 5 days re-exposure. The timeline of the study, which to our knowledge has not been tested before, could then have differentially affected these biochemical outcomes. Despite large variability, this evidence suggests differential neuroadaptive alterations in distinct mouse strains, highlighting the need for further investigations, which could focus on probing the spatiotemporal patterns of neuromodulatory signals in the brain during behavior.

Exploratory measurements revealed significant cation imbalances in the cerebellum of C3H and B6 alcohol-exposed mice. In agreement with a previous report in DBA/2J male mice, C3H alcohol-exposed mice displayed trends toward hyponatremia and perhaps hypokalemia (Belknap et al., 1978). In contrast, B6 alcohol-exposed mice displayed higher levels of Mg^++^. Several clinical studies described hyponatremia, hypomagnesemia and hypokalemia in plasma or serum of AUD patients, both during chronic alcohol exposure and withdrawal (Te Riele and Verrips, 2014; Baj et al., 2020). It was also suggested that alcohol increases intracellular Ca^++^ by upregulation of voltage-gated Ca^++^ channels, inhibition of Ca^++^ extrusion, and Mg^++^ depletion (Husain et al., 2014). However, there have been contrasting evidence on Ca^++^ levels following alcohol exposure (Skalny et al., 2016; Baj et al., 2020). Alcohol-induced changes in electrolyte signaling may interfere with neuronal homeostasis and neural circuit function, and further research is required to fully understand their role in AUD.

The typical oral route of alcohol exposure used in humans, was chosen for this study. The liquid diets containing alcohol caused mice to gain weight more slowly than controls, but in a comparable way between the two strains and it is thus unlikely to cause any strain differences. A potential confounder for the more pronounced alcohol withdrawal symptoms observed in the B6 mice is the higher alcohol intake of the B6 mice compared to the C3H mice, although BACs were comparable. Another limitation of the study is that BACs, measured at the end of the experiment, did not reach the expected values previously described (Bornebusch et al., 2021). Terminal blood sampling was performed 2–4 h after last exposure, allowing the mice to be in the acute withdrawal phase and collection of all relative brain samples. The time interval likely allowed BAC to decrease in the mice sampled last, leading to increased variability and underestimated BAC. While low levels could be an artifact due to measuring the BACs at a single time point and being dependent on the feeding routine, low BACs could also have played a significant role in the relatively weak withdrawal symptoms and biochemical effects. Therefore, they should be considered when examining the data as well as for future experiments. In this regard, exposing the mice to alcohol vapors would ensure more constant and consistent alcohol exposure.

The series of behavioral and biochemical tests was chosen to cover the multi-domain aspects of alcohol withdrawal, while minimizing stress to the animals, and could be further optimized. While most of these behavioral endpoints have been previously described as part of a test series in other mouse strains, here we extended the comparison to neurochemical endpoints, including neuroinflammatory markers, as well as USVs and cations measurements (Crabbe et al., 2005; Metten et al., 2018). Additionally, beside investigating the long-term alcohol withdrawal symptoms, we also explored the effects of re-exposure on the neurochemical mechanisms. In conclusion, our initial feasibility study suggests that the commercially available inbred mouse B6 strain is more suitable than the C3H strain to study the long-term effects of alcohol withdrawal, and that alcohol vapor exposure should be used for future investigations.

## Data availability statement

The raw data supporting the conclusions of this article will be made available by the authors, without undue reservation.

## Ethics statement

The animal study was reviewed and approved by Animal Experiments Inspectorate under the Danish Ministry of Food, Agriculture, and Fisheries.

## Author contributions

ST and MT conceptualized the studies, designed the experiments, analyzed the data, and interpreted the data. ST collected the all behavioral data. ST and PW collected the all biochemical data. ST and TB collected the neuroinflammatory data. ST and MT wrote the manuscript with important input from PW and TB. All authors contributed to the article and approved the submitted version.

## Supplementary material

Supplementary Figure 1 No significant depressive-like effects of alcohol exposure and subsequent withdrawal in the forced swim test. The % of time spent immobile **(A,B)** and the latencies in seconds to first immobility **(C,D)** were assessed in C3H and B6 mice at withdrawal day 8. Data are shown as individual subjects, with violin plots representing group means, distribution and density **(A,B)**. Latencies are shown as the % of mice showing a response as a function of time **(C,D)**. Note the truncated ordinate in panel **(A)** and truncated abscissa in panel **(D)**. *n* = 6 per group/strain.

Supplementary Figure 2 No significant anxiogenic-like effects of alcohol exposure and subsequent withdrawal in the elevated zero maze. The % of time spent in the open arms **(A,B)**, the number of entries into open arms **(C,D)**, the number of SAPs **(E,F)** and the latencies in seconds to first entry into an open arm **(G,H)** were assessed in C3H and B6 mice at withdrawal day 19. Data are shown as individual subjects with violin plots **(A–F)**. Latencies are shown as the % of mice showing a response as a function of time **(G,H)**. Note the truncated ordinate in panels **(B,C,F)**, and the truncated abscissa in panel H. *n* = 12 per group/strain.
