# Supplementary figures and images for: Behavioral and biochemical effects of alcohol withdrawal in female C3H/HeNRj and C57BL/6JRj mice
Source: Front Behav Neurosci. 2023 Feb 23;17:1143720. doi: 10.3389/fnbeh.2023.1143720 (PMC9995974; doi:10.3389/fnbeh.2023.1143720)

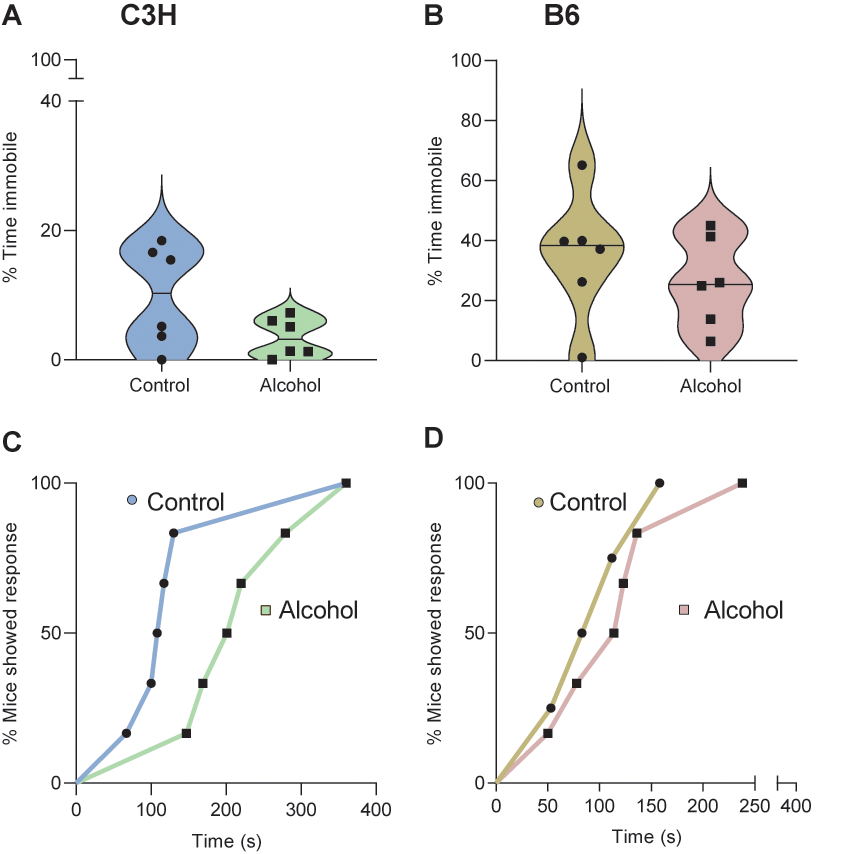

Supplement: Supplementary Figure 1 — No significant depressive-like effects of alcohol exposure and subsequent withdrawal in the forced swim test. The % of time spent immobile (A,B) and the latencies in seconds to first immobility (C,D) were assessed in C3H and B6 mice at withdrawal day 8. Data are shown as individual subjects, with violin plots representing group means, distribution and density (A,B). Latencies are shown as the % of mice showing a response as a function of time (C,D). Note the truncated ordinate in panel (A) and truncated abscissa in panel (D). n = 6 per group/strain. [file Image_1.TIF]

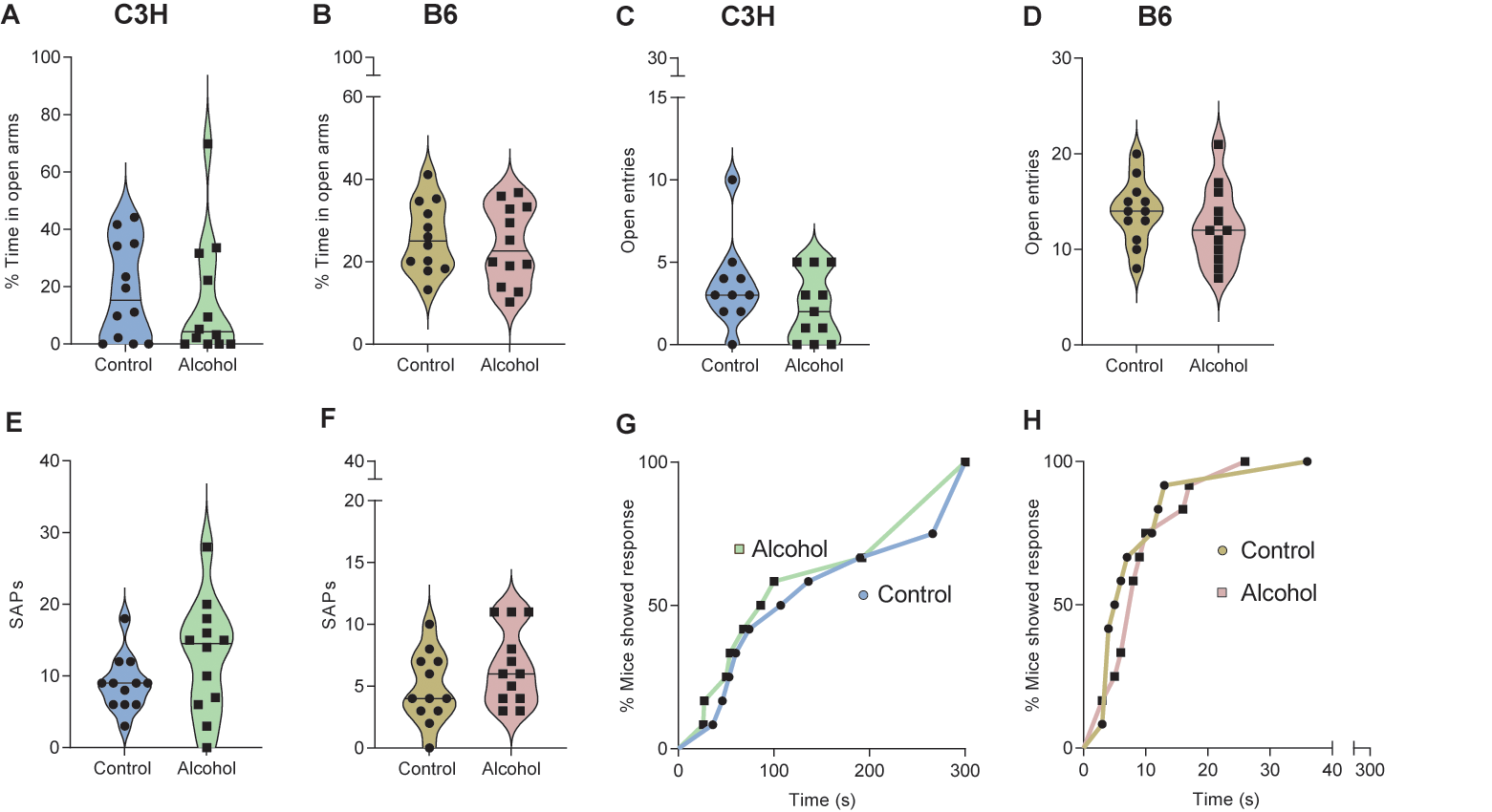

Supplement: Supplementary Figure 2 — No significant anxiogenic-like effects of alcohol exposure and subsequent withdrawal in the elevated zero maze. The % of time spent in the open arms (A,B), the number of entries into open arms (C,D), the number of SAPs (E,F) and the latencies in seconds to first entry into an open arm (G,H) were assessed in C3H and B6 mice at withdrawal day 19. Data are shown as individual subjects with violin plots (A–F). Latencies are shown as the % of mice showing a response as a function of time (G,H). Note the truncated ordinate in panels (B,C,F), and the truncated abscissa in panel H. n = 12 per group/strain. [file Image_2.TIF]
